# Enterocutaneous Fistula: A Challenging Complication in Cervical Cancer Management

**DOI:** 10.7759/cureus.87475

**Published:** 2025-07-07

**Authors:** Abigayle Wyer, Mena Louis, Oluwasemilore I Okunlola, Raven Richardson, Timothy J Stevens

**Affiliations:** 1 Surgery, Northeast Georgia Medical Center Gainesville, Gainesville, USA; 2 General Surgery, Northeast Georgia Medical Center Gainesville, Gainesville, USA; 3 General Surgery, St. George's University School of Medicine, True Blue, GRD; 4 Trauma and Acute Care Surgery, Northeast Georgia Medical Center Gainesville, Gainesville, USA

**Keywords:** anti-angiogenic therapy, bevacizumab, cervical cancer, chemotherapy complications, enterocutaneous fistula, fistula, pelvic abscess

## Abstract

A 42-year-old woman with a history of cervical cancer previously treated with radical hysterectomy, lymphadenectomy, and bilateral salpingectomy presented with severe right lower extremity pain, initially attributed to routine chemotherapy-related side effects. Despite symptomatic management, her pain intensified, accompanied by abdominal distention, constipation, and reduced ambulation. Subsequent clinical evaluation revealed a significant abscess extending from the pelvis into the gluteal musculature and thigh. Imaging confirmed that the abscess communicated with a loop of the small bowel, evolving into an enterocutaneous fistula, which manifested as feculent drainage through her thigh incision. Surgical intervention with incision, drainage, and excision of nonviable muscle tissue provided temporary relief but could not entirely resolve the fistula. Microbiological cultures yielded intestinal flora, including *Escherichia coli*, *Enterococcus faecium*, and *Bacteroides ovatus*, confirming bowel origin. A thorough review of the patient's clinical history identified bevacizumab therapy as a significant risk factor, consistent with current evidence linking bevacizumab use to increased fistula formation, especially following prior pelvic surgery or radiation therapy. Bevacizumab, an anti-angiogenic monoclonal antibody that inhibits vascular endothelial growth factor, disrupts essential physiological processes of angiogenesis and tissue repair, thereby facilitating fistula development in susceptible tissues. Management employed conservative measures, including nutritional support, infection control, and wound management using an external ostomy appliance, ultimately achieving medical stabilization. Bevacizumab therapy carries a significant risk of severe complications, including fistula formation, particularly in patients with prior pelvic surgery or radiation therapy. Early clinical suspicion, prompt imaging studies, and a coordinated multidisciplinary approach are essential for timely diagnosis and effective management of these complications. Patients receiving bevacizumab should undergo careful monitoring, especially if they have undergone previous pelvic treatments, to allow for prompt detection and intervention of potential fistula formation.

## Introduction

Cervical cancer remains a significant global health issue, representing one of the leading malignancies affecting women worldwide [1]. Although the incidence has declined in countries with effective screening programs, cervical cancer continues to cause substantial morbidity and mortality, especially in populations with limited healthcare access [2]. Treatment for cervical cancer typically involves surgery, radiation, chemotherapy, or a combination of these modalities, depending on the stage and extent of the disease [3]. In advanced or recurrent cervical cancer cases, targeted therapy has emerged as an important adjunct, enhancing survival rates while aiming to maintain the quality of life.

Bevacizumab, a monoclonal antibody targeting vascular endothelial growth factor (VEGF), has significantly impacted the treatment landscape for advanced cervical cancer [4]. Its use, particularly in combination with chemotherapy, has demonstrated improved survival outcomes in patients with recurrent, persistent, or metastatic disease [5]. By inhibiting angiogenesis, bevacizumab disrupts tumor blood supply, thereby impairing growth and metastatic potential [6]. However, its mechanism of action carries inherent risks due to the critical role of VEGF in normal tissue healing and repair processes [7]. Consequently, bevacizumab therapy has been associated with notable adverse effects, including hypertension, proteinuria, hemorrhage, thromboembolic events, and delayed wound healing [8].

Among the serious complications associated with bevacizumab therapy, fistula formation has increasingly garnered attention in clinical oncology [8]. Fistulas, abnormal communications between two epithelial-lined organs or between an organ and the skin, significantly impair patient quality of life and pose substantial therapeutic challenges [9]. This complication arises more frequently in patients treated with bevacizumab, particularly when therapy follows surgery or radiotherapy [10]. The mechanism behind increased fistula formation involves bevacizumab-induced inhibition of angiogenesis, which impairs tissue healing, reduces vascular integrity, and ultimately facilitates fistulous communication through weakened tissue planes [11].

Multiple studies have documented elevated rates of gastrointestinal and genitourinary fistulas among patients receiving bevacizumab in the context of pelvic malignancies, notably cervical and colorectal cancers [12]. The increased risk is especially prominent in those who have undergone pelvic surgery or radiotherapy due to compromised vascular supply and tissue healing capacity in these previously treated regions [13]. Awareness of this complication is essential for timely diagnosis and management, as early detection and intervention may significantly reduce patient morbidity and healthcare burden.

## Case presentation

A 42-year-old woman with a history of cervical cancer treated initially with radical abdominal hysterectomy, lymphadenectomy, bilateral salpingectomy, and ovarian transposition in November 2021 presented to the emergency department with severe right leg and abdominal pain. Her cervical cancer had been complicated by a likely localized recurrence around her right ureter, for which she was undergoing chemotherapy. After her second chemotherapy session, she reported worsening right upper leg pain, initially attributed by her oncology team to muscular side effects. Despite the reassurance, the pain progressed, involving her right hip and rendering her unable to ambulate or abduct her right hip. She also experienced abdominal distention and constipation, and reported having no significant bowel movements for approximately one week despite attempting suppositories.

**Figure 1:**
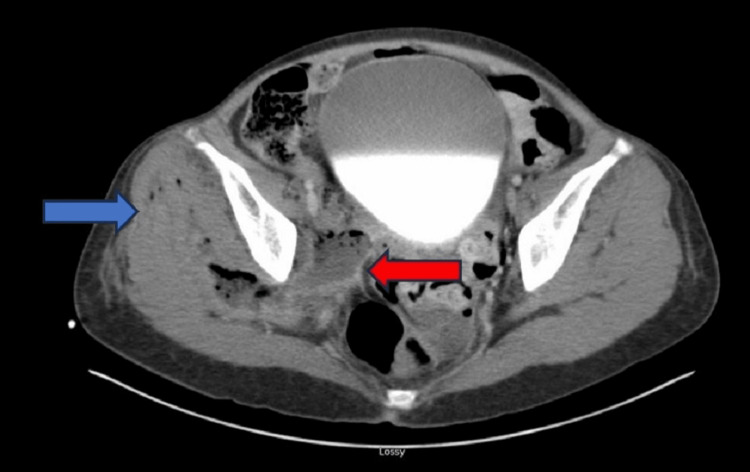
Axial CT image of the pelvis demonstrating a complex pelvic abscess characterized by fluid and gas collections in the right hemipelvis (blue arrow), extending into the gluteal musculature. Notably, the abscess shows a clear communication with an adjacent loop of small bowel (red arrow) CT: computed tomography

**Figure 2:**
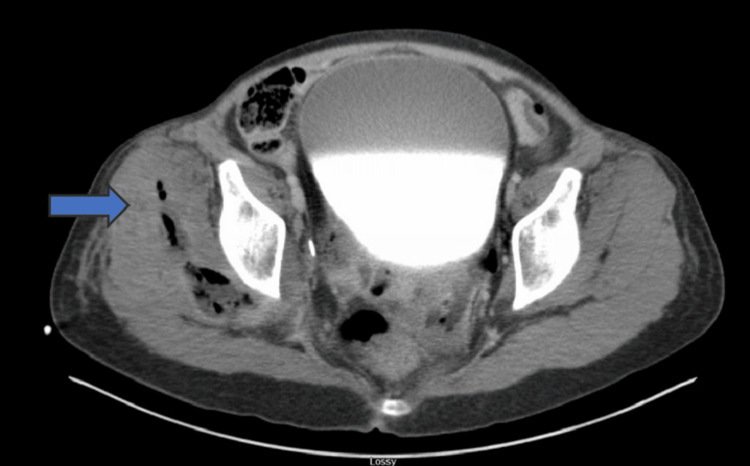
Axial CT image of the pelvis demonstrating a complex pelvic abscess characterized by fluid and gas collections in the right hemipelvis (blue arrow), extending into the gluteal musculature CT: computed tomography

**Figure 3:**
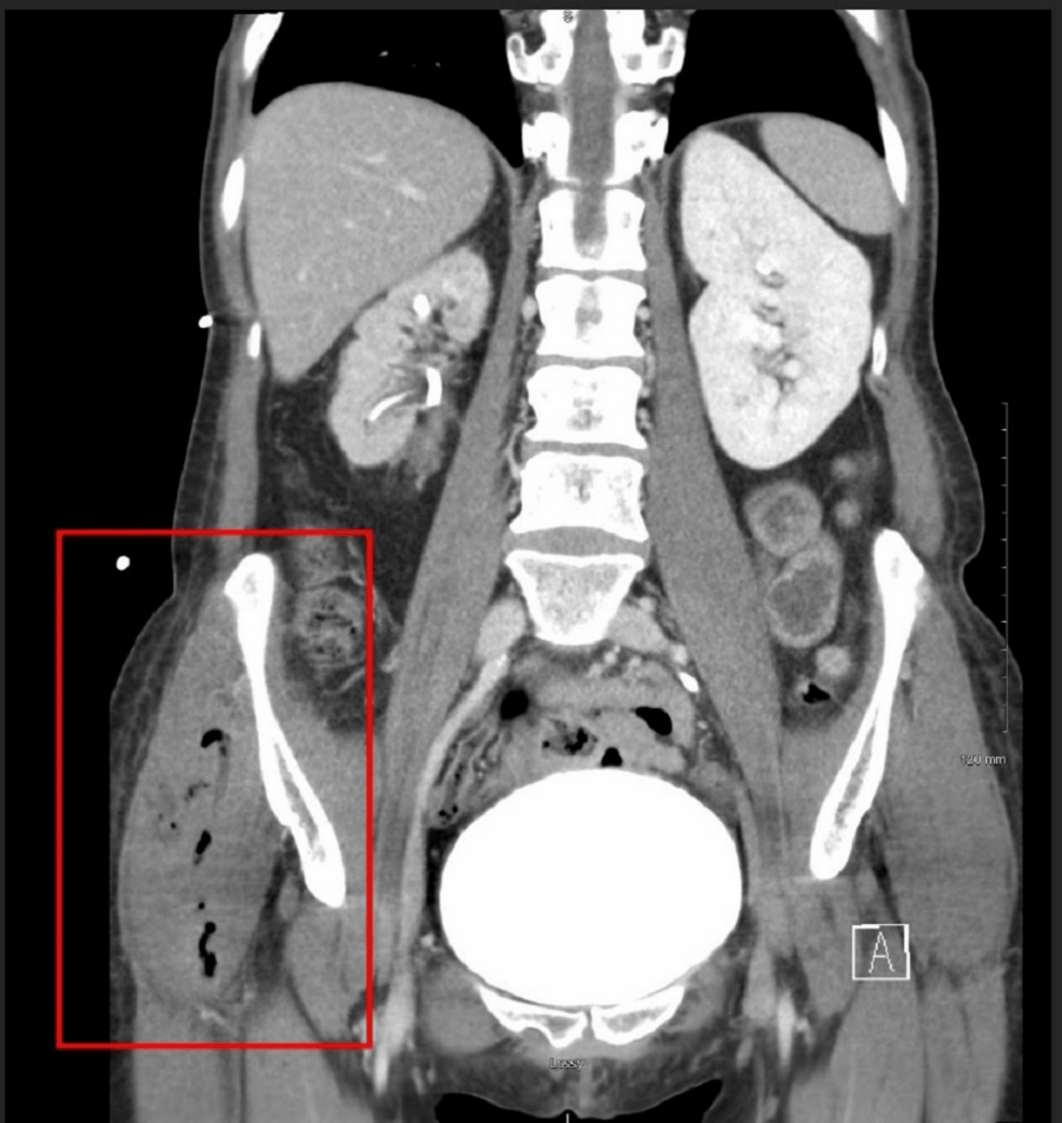
Coronal CT image of the abdomen and pelvis illustrating a prominent area of fluid and gas collection within the right hemipelvis indicating a pelvic abscess (marked by a red rectangle) CT: computed tomography

Initial laboratory evaluation revealed mild anemia, normal leukocyte counts, and a normal platelet count. The basic metabolic profile and inflammatory markers were within acceptable limits, except for a mildly elevated alkaline phosphatase (Table 1). Imaging studies performed upon admission demonstrated a complex abscess with fluid and gas collections in the right hemipelvis, extending into the gluteal musculature and communicating with a loop of small bowel. Subsequent cultures from the fluid obtained during the operative intervention grew *Bacteroides ovatus*, *Escherichia coli*, and *Enterococcus faecium*, indicating an intestinal origin. A CT angiography of the lower extremity confirmed significant obturator and gluteal myositis with associated gas and fluid collections, raising concern for a developing enterocutaneous fistula (Figures 1-3).

**Table 1:** Relevant laboratory values on admission

Lab	Value	Reference value
Hemoglobin	10.6 g/dL	12.0-15.5 g/dL
Hematocrit	32.3%	36-48%
White blood cell count	9.7 x 103/µL	4,000-11,000 µL
Platelets	193 x 103/µL	150,000-450,000 µL
Alkaline phosphatase	122 IU/L	44-147 IU/L

**Figure 4:**
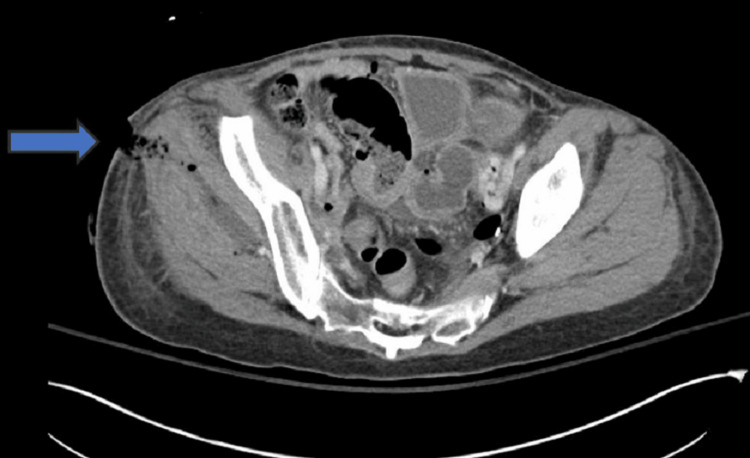
Axial CT image of the pelvis demonstrating progression of the pelvic fluid collection with clear evidence of a fistulous connection between the bowel and the surgical wound. The blue arrows indicate the fistula tract CT: computed tomography

**Figure 5:**
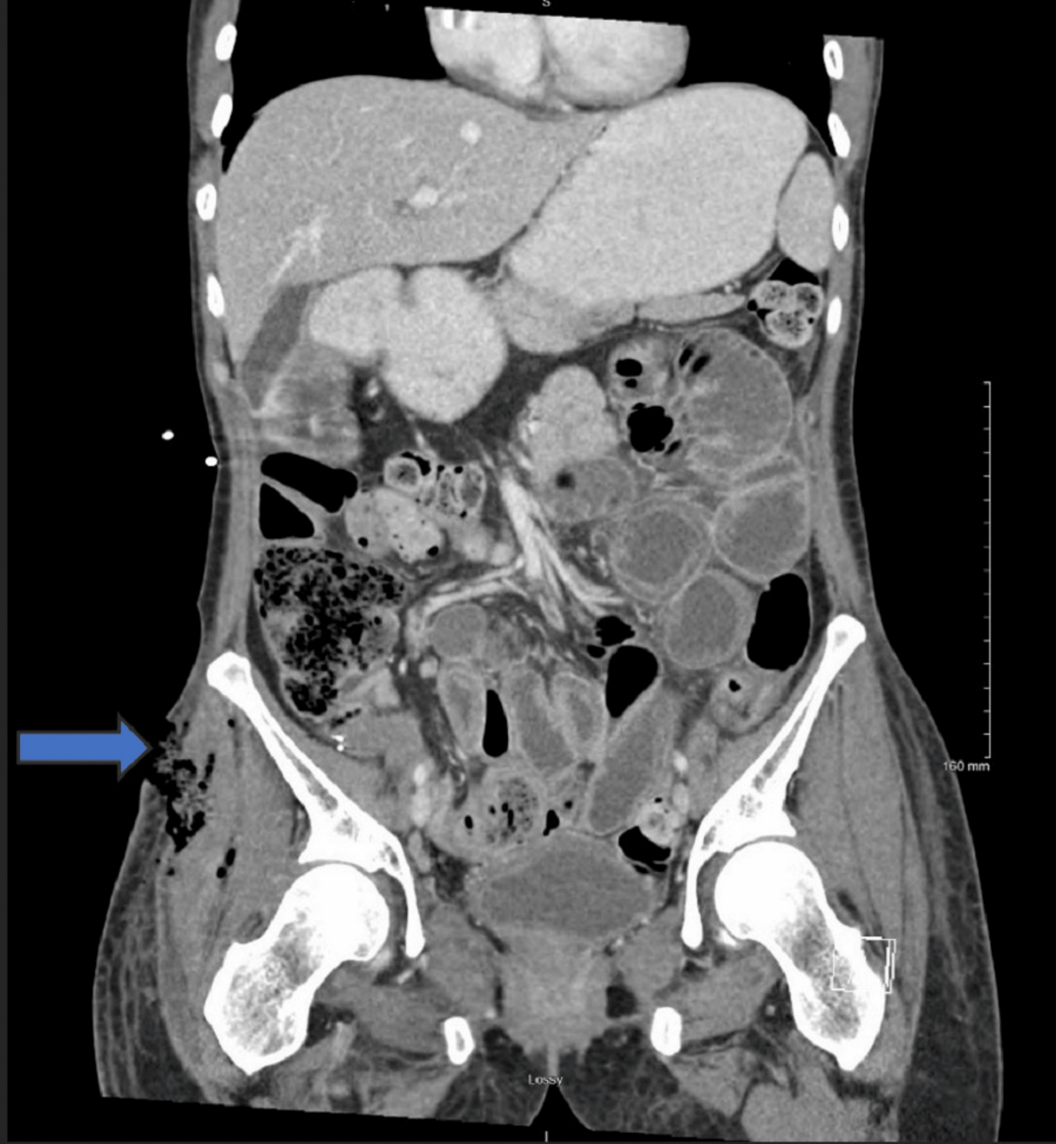
Coronal CT image of the pelvis demonstrating progression of the pelvic fluid collection with clear evidence of a fistulous connection between the bowel and the surgical wound. The blue arrows indicate the fistula tract CT: computed tomography

**Figure 6:**
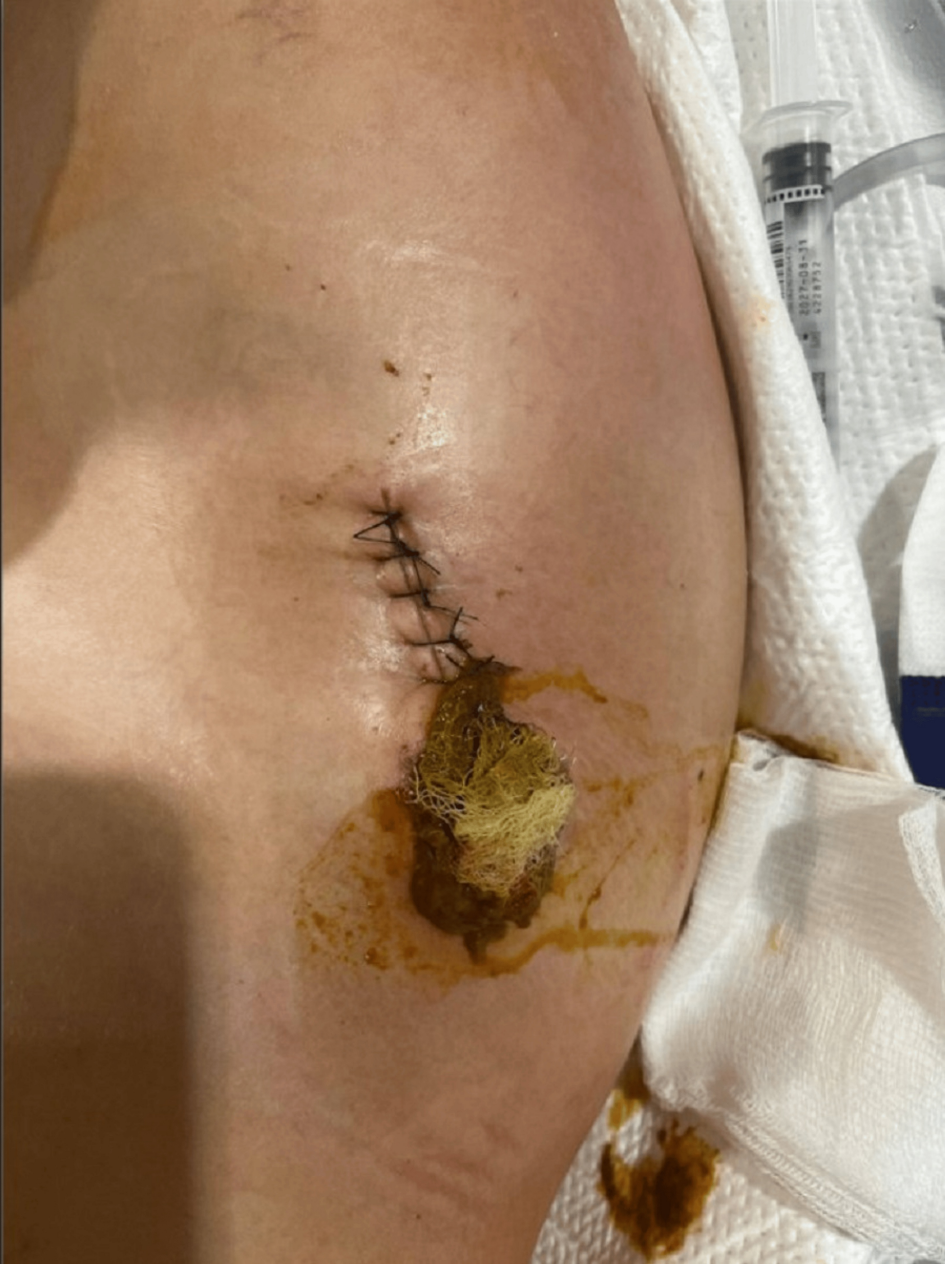
Clinical photograph showing the surgical wound site on postoperative day 3 with foul-smelling feculent drainage consistent with enterocutaneous fistula output

The patient was admitted to the acute care surgical service and underwent urgent surgical incision and drainage of the right hip and thigh abscess on hospital day two. Intraoperative findings included extensive subcutaneous edema and multiple loculated pockets of foul-smelling purulent fluid within the muscle compartments, necessitating excision of approximately 4 cm² of nonviable muscle tissue. Despite adequate drainage and initial stabilization, postoperative imaging demonstrated the progression of the fluid collection. It confirmed the presence of a direct fistulous communication between the bowel and the surgical wound, manifesting clinically as feculent drainage from the surgical site (Figures 4-6).

**Figure 7:**
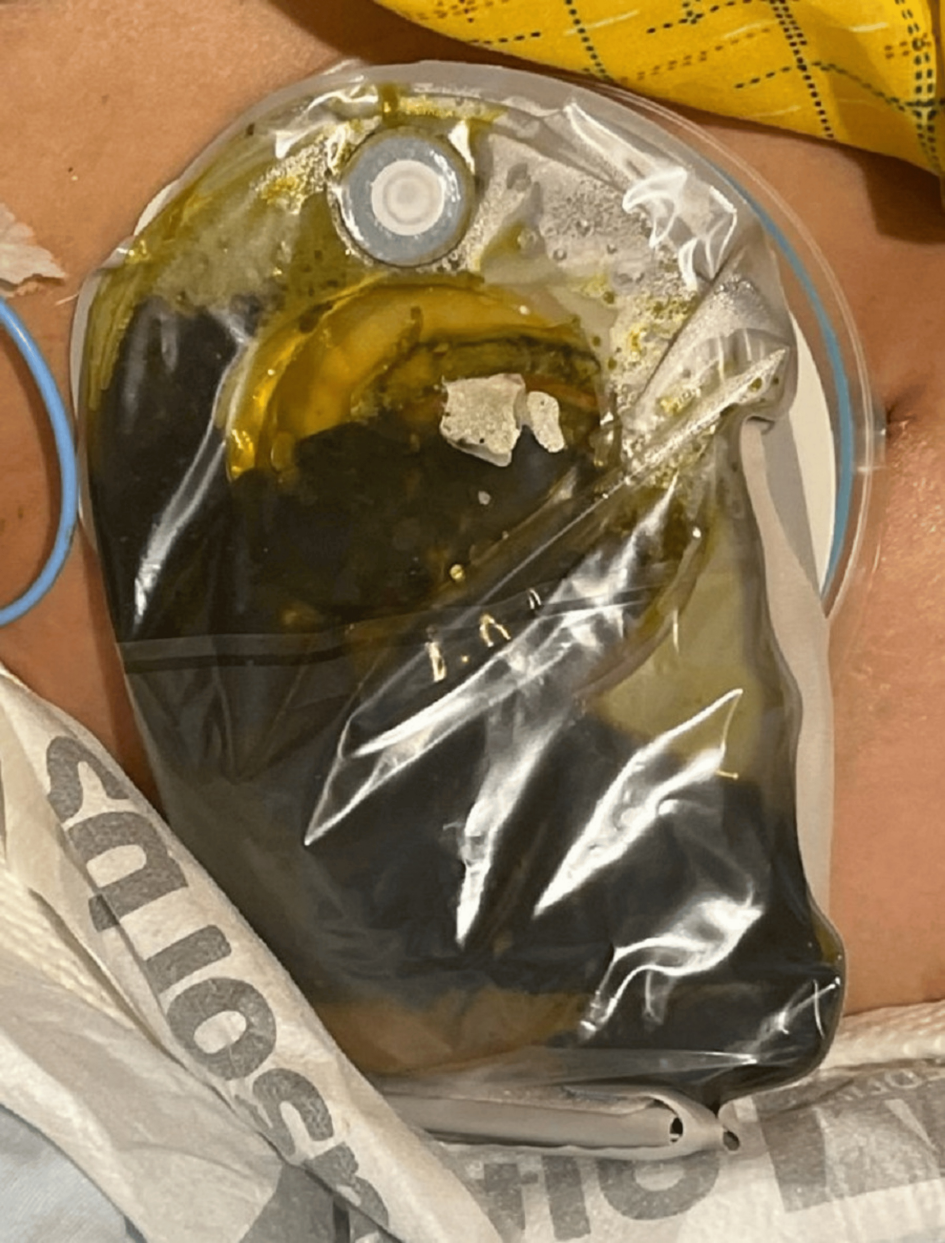
Clinical photograph on postoperative day 4 showing the fistula managed with an ostomy appliance capturing significant stool output from the wound site

**Figure 8:**
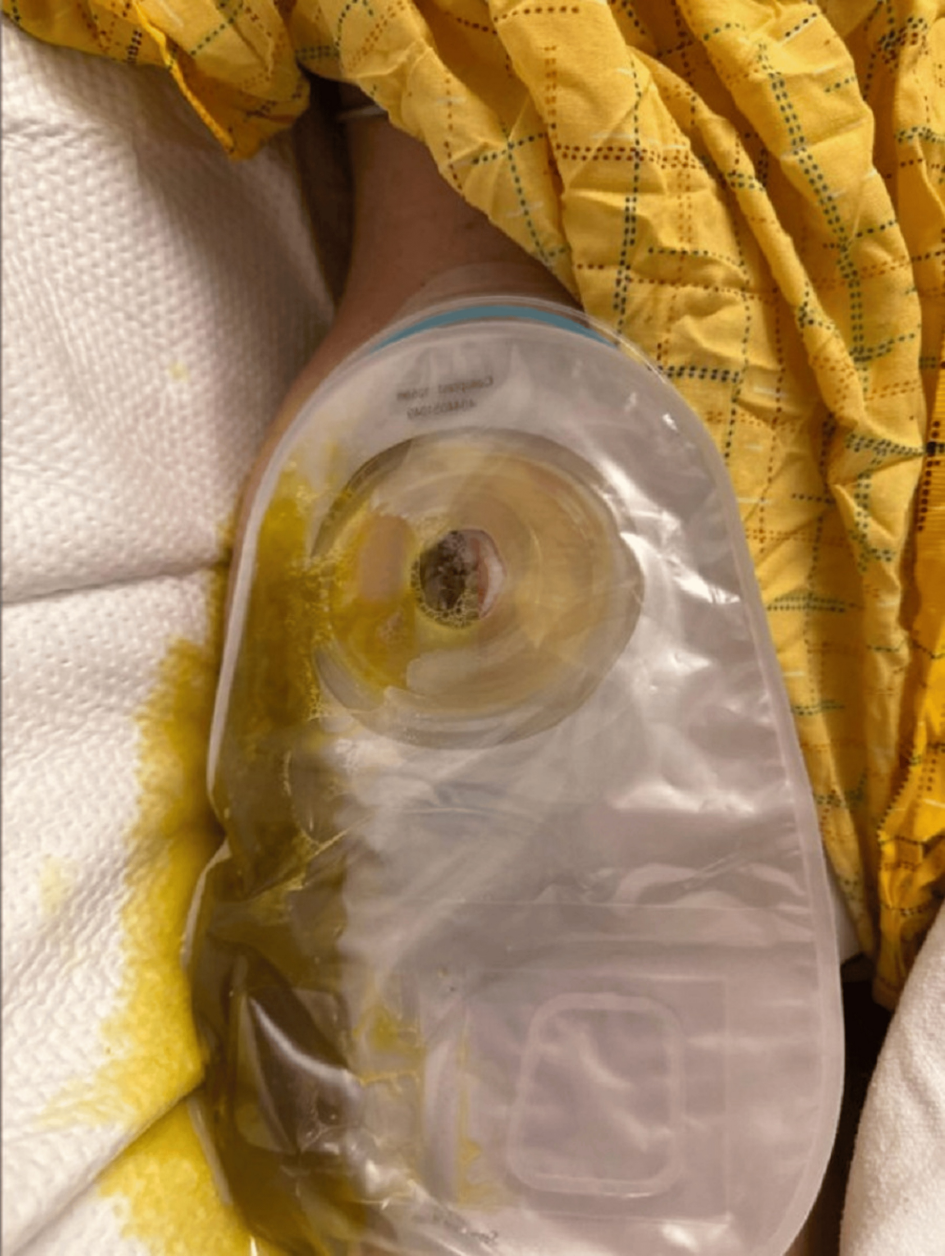
Clinical photograph on postoperative day 18 demonstrating ongoing management of the fistula with an ostomy appliance with continued fistula output

During hospitalization, the patient developed an overt enterocutaneous fistula, with significant stool output draining through the thigh incision site, requiring management with an external ostomy appliance (Figures 7-8). Persistent abdominal cramps and partial small bowel obstruction further complicated her clinical course. Conservative management was initially pursued to optimize nutritional status and promote healing, but the patient required ongoing analgesic support and wound care. Ultimately, medical stabilization was achieved through supportive measures. She demonstrated gradual improvement and was eventually discharged with ongoing wound management instructions, nutritional support, and close outpatient follow-up.

## Discussion

Unusual wounds in typical anatomical locations present a diagnostic challenge, especially when they fail to respond to conventional treatments [14]. Chronic or non-healing wounds require clinicians to maintain a broad differential diagnosis, considering possibilities such as underlying infection, malignancy, foreign bodies, immunocompromised states, and, notably, the potential presence of a fistula. Enterocutaneous fistulas, abnormal connections between the gastrointestinal tract and skin, are particularly important to recognize early, as delayed diagnosis significantly increases morbidity and complicates management [15]. Clinicians should suspect fistula formation in any chronic wound exhibiting persistent drainage of fluid with unusual odor, feculent material, gas bubbles, or unexplained skin breakdown despite standard wound care.

When evaluating a patient with a chronic, non-healing wound, detailed patient history and thorough clinical examination are fundamental. A history of previous abdominal or pelvic surgery, radiation therapy, malignancy, inflammatory bowel disease, or recent chemotherapy raises suspicion for an enterocutaneous fistula. Clinical examination findings suggestive of a fistulous communication include persistent drainage, skin irritation, unusual discharge color, and gaseous emissions [16]. Prompt diagnostic imaging, typically contrast-enhanced CT, helps delineate fistulous tracts, localize the source of communication, and identify associated complications such as abscess formation or bowel obstruction. Early imaging has a significant impact on management strategies and patient outcomes.

Management of enterocutaneous fistulas begins with stabilization of the patient, control of sepsis, nutritional optimization, and detailed assessment of wound output and characteristics [17]. Conservative management typically involves initial non-operative strategies, including intravenous antibiotics, nutritional support (often parenteral nutrition), and wound care aimed at controlling drainage and protecting surrounding skin integrity. An ostomy appliance can be used effectively for skin protection and fluid containment, thereby improving patient comfort and quality of life. Non-operative management is particularly valuable early in the clinical course, allowing the fistula to close spontaneously if conditions are optimal, typically within several weeks to months [18].

However, surgical intervention becomes necessary in patients who fail conservative management, exhibit persistent high-output fistulas, uncontrolled sepsis, or progressive nutritional deterioration [19]. The optimal timing of surgical repair is critical, with literature supporting delayed surgical approaches to allow inflammation to subside, nutritional status to improve, and infection to be adequately controlled [19]. Surgical intervention typically involves resection of involved bowel segments, careful closure or diversion of the fistula, and meticulous handling of tissue to promote healing. Surgical decision-making requires individualized assessment, balancing risks associated with immediate intervention against the potential morbidity of prolonged conservative management.

In the context of cervical cancer treatment, chemotherapy, and specifically anti-angiogenic therapy such as bevacizumab, significantly increases the risk of fistula formation, especially in previously irradiated or surgically altered pelvic tissues [5]. Anti-angiogenic agents compromise normal vascular repair mechanisms, impairing wound healing and increasing tissue susceptibility to fistula formation [4]. Recognizing this elevated risk enables clinicians to closely monitor these patients, ensuring early intervention at initial signs of fistula formation, thereby preventing severe complications.

A multidisciplinary approach remains essential for managing complex wounds complicated by fistula formation. Coordinated efforts involving surgeons, wound care specialists, infectious disease experts, dietitians, and oncologists to optimize patient outcomes. Patient education, emphasizing the importance of reporting persistent or unusual symptoms, also plays a significant role.

Patients experiencing chronic wounds complicated by fistula formation often endure significant psychological distress due to prolonged hospitalizations, physical discomfort, altered body image, and impaired social functioning. Addressing the mental health of these patients is essential and requires a compassionate, empathetic approach involving early psychological assessment, emotional support, and counseling services. Establishing open communication, setting realistic expectations, and involving patients actively in decision-making can help restore their sense of autonomy and control. Additionally, integrating mental health professionals into the multidisciplinary team and offering consistent emotional support through peer groups or counseling can significantly improve patients' coping mechanisms, adherence to treatment, and overall quality of life.

## Conclusions

This case report emphasizes the significant risk of fistula formation associated with bevacizumab therapy in cervical cancer patients, particularly in those previously treated with pelvic surgery or radiotherapy. Clinicians must remain vigilant, maintaining a high level of suspicion for early symptoms suggestive of this complication. Early identification, timely imaging, and a coordinated multidisciplinary approach are vital to managing these complex clinical situations effectively, thereby minimizing morbidity and improving patient outcomes.

## References

[1] Zhang S, Xu H, Zhang L, Qiao Y (2020). Cervical cancer: epidemiology, risk factors and screening. Chin J Cancer Res.

[2] Castle PE (2024). Looking back, moving forward: challenges and opportunities for global cervical cancer prevention and control cervical cancer management. Viruses.

[3] Burmeister CA, Khan SF, Schäfer G, Mbatani N, Adams T, Moodley J, Prince S (2022). Cervical cancer therapies: current challenges and future perspectives. Tumour Virus Res.

[4] Bizzarri N, Ghirardi V, Alessandri F (2016). Bevacizumab for the treatment of cervical cancer. Expert Opin Biol Ther.

[5] Tewari KS, Sill MW, Penson RT (2017). Bevacizumab for advanced cervical cancer: final overall survival and adverse event analysis of a randomised, controlled, open-label, phase 3 trial (Gynecologic Oncology Group 240). Lancet.

[6] Lorusso D, Colombo N, Dubot C (2025). Pembrolizumab plus chemotherapy for advanced and recurrent cervical cancer: final analysis according to bevacizumab use in the randomized KEYNOTE-826 study. Ann Oncol.

[7] Kotaka S, Kondo E, Kawai Y (2023). Real-world efficacy and safety of bevacizumab single-maintenance therapy following platinum-paclitaxel chemotherapy plus bevacizumab in patients with advanced cervical cancer. J Gynecol Oncol.

[8] Sugiyama T, Katsumata N, Toita T, Ura M, Shimizu A, Kamijima S, Aoki D (2022). Incidence of fistula occurrence in patients with cervical cancer treated with bevacizumab: data from real-world clinical practice. Int J Clin Oncol.

[9] Tuma F, Crespi Z, Wolff CJ, Daniel DT, Nassar AK (2020). Enterocutaneous fistula: a simplified clinical approach. Cureus.

[10] Ganapathi AM, Westmoreland T, Tyler D, Mantyh CR (2012). Bevacizumab-associated fistula formation in postoperative colorectal cancer patients. J Am Coll Surg.

[11] Eriksen M, Bulut O (2014). Chemotherapy-induced enterocutaneous fistula after perineal hernia repair using a biological mesh: a case report. Int Med Case Rep J.

[12] Yang ST, Liu HH, Liu CH, Wang LW, Wang PH (2024). Bevacizumab is associated with a higher gastrointestinal/genitourinary fistula or perforation risk in cervical cancer patients undergoing pelvic radiotherapy. Int J Gynaecol Obstet.

[13] Sfakianos GP, Numnum TM, Halverson CB, Panjeti D, Kendrick JE 4th, Straughn JM Jr (2009). The risk of gastrointestinal perforation and/or fistula in patients with recurrent ovarian cancer receiving bevacizumab compared to standard chemotherapy: a retrospective cohort study. Gynecol Oncol.

[14] Pozez AL, Aboutanos SZ, Lucas VS (2007). Diagnosis and treatment of uncommon wounds. Clin Plast Surg.

[15] Wolny D, Štěpánek L, Horáková D, Thomas J, Zapletalová J, Patel MS (2024). Risk factors for non-healing wounds-a single-centre study. J Clin Med.

[16] Gefen R, Garoufalia Z, Zhou P, Watson K, Emile SH, Wexner SD (2022). Treatment of enterocutaneous fistula: a systematic review and meta-analysis. Tech Coloproctol.

[17] Owen RM, Love TP, Perez SD (2013). Definitive surgical treatment of enterocutaneous fistula: outcomes of a 23-year experience. JAMA Surg.

[18] Gribovskaja-Rupp I, Melton GB (2016). Enterocutaneous fistula: Proven strategies and updates. Clin Colon Rectal Surg.

[19] Lee SH (2012). Surgical management of enterocutaneous fistula. Korean J Radiol.

